# Differential susceptibility of Dectin-1 isoforms to functional inactivation by neutrophil and fungal proteases

**DOI:** 10.1096/fj.201701145R

**Published:** 2018-01-22

**Authors:** James S. Griffiths, Aiysha Thompson, Matthew Stott, Ankita Benny, Natalie A. Lewis, Philip R. Taylor, Julian Forton, Sarah Herrick, Selinda J. Orr, Eamon P. McGreal

**Affiliations:** * Division of Infection and Immunity, Cardiff University School of Medicine, Cardiff, United Kingdom;; † School of Medicine, Cardiff University School of Medicine, Cardiff, United Kingdom;; ‡ Children’s Hospital for Wales, Cardiff, United Kingdom;; § School of Biological Sciences, Faculty of Biology Medicine and Health, Manchester Academic Health Science Centre, University of Manchester, Manchester, United Kingdom;; ¶ Centre for Medical Education, Cardiff University School of Medicine, Cardiff, United Kingdom

**Keywords:** elastase, C-type lectin-like receptor, cystic fibrosis, *Aspergillus*

## Abstract

Patients with cystic fibrosis (CF) experience chronic or recurrent bacterial and fungal lung infections. Many patients with CF cannot effectively clear *Aspergillus* from their lungs. This may result in IgE sensitization and the development of allergic bronchopulmonary aspergillosis, or invasive infections, such as *Aspergillus* bronchitis. Lung disease in patients with CF is associated with neutrophil-dominated inflammation and elevated levels of the serine protease, neutrophil elastase (NE). Various C-type lectin-like receptors (CLRs), including Dectin-1 and Dectin-2, are involved in the immune response to *Aspergillus*. Here, we show that purified NE cleaves Dectin-1 in an isoform-specific manner. Bronchoalveolar lavage fluid from patients with CF, which contains high NE activity, induces Dectin-1 cleavage. Similarly, filtrate from a protease-producing strain of *Aspergillus fumigatus* induces isoform-specific cleavage of Dectin-1. Dectin-1 knockout (KO) cells and NE-treated cells demonstrated reduced phagocytosis of zymosan, a fungal cell wall preparation. In addition, NE cleaves 2 other CLRs, Dectin-2 and Mincle, and fungal-induced cytokine production was reduced in Dectin-1 KO cells, Dectin-2 KO cells, and NE-treated cells. Thus, Dectin-1 and Dectin-2 cleavage by NE and/or *A. fumigatus–*derived proteases results in an aberrant antifungal immune response that likely contributes to disease pathology in patients with CF.—Griffiths, J. S., Thompson, A., Stott, M., Benny, A., Lewis, N. A., Taylor, P. R., Forton, J., Herrick, S., Orr, S. J., McGreal, E. P. Differential susceptibility of Dectin-1 isoforms to functional inactivation by neutrophil and fungal proteases.

*Aspergillus fumigatus* has long been recognized as a serious invasive pathogen in immunocompromised patients and carries a high mortality rate. More recently, it has increasingly been acknowledged as an important pathogen in patients with preexisting airway disease who are otherwise immunocompetent. Common diseases, including asthma, chronic obstructive pulmonary disease, and cystic fibrosis (CF), are frequently complicated by this fungus (1). The spectrum of disease caused by *A. fumigatus* in these patients includes invasive infection and allergic hypersensitivity, both of which cause progressive lung disease. Worldwide, the number of patients with underlying airway disease who are affected by this fungus exceeds 10 million (1). To elicit disease, the fungus must first access and persist in the airway. The normally functioning host is well adapted to prevent such persistence; however, *A. fumigatus* is isolated in 10–57% of airway secretions from patients with CF (2). *A. fumigatus*–specific IgG has also been detected in 41% of patients with CF by age 4 yr, increasing to 98% by age 10 yr. These seropositivity rates were significantly higher than either control or asthmatic patients (3). Allergic bronchopulmonary aspergillosis (ABPA), a serious allergic entity that is characterized by airway obstruction and irreversible bronchiectasis, affects 17.7% of the adult CF population, whereas *Aspergillus* bronchitis is present in 30% of patients (4). A central question, therefore, is why patients with underlying airway disease are susceptible to *A. fumigatus*.

In addition to the critical action of the mucocilliary escalator in clearing pathogens and other foreign particles from the airway, the immune system takes a multifaceted approach to the recognition and elimination of pathogenic fungi. TLRs and C-type lectin-like receptors (CLRs) expressed by myeloid cells are essential components of these responses (5). Macrophages from mice that lack functional TLR2, TLR4, or both receptors displayed defective inflammatory responses to *A. fumigatus* (6). In addition, a study that examined single nucleotide polymorphisms (SNPs) in TLR2, TLR3, TLR4, and TLR9 identified an association between a TLR4 haplotype and an increased risk of developing invasive aspergillosis in a cohort of patients who underwent hematopoietic stem cell transplantation (7). Immunocompetent mice that are deficient in the CLR, Dectin-1, are exquisitely sensitive to *A. fumigatus* airway infection, with an 80% mortality rate (8). The β-glucan receptor, Dectin-1, binds specific morphologies of *A. fumigatus*, including swollen conidia, early germlings, and hyphae, but not resting conidia (9, 10). Dectin-1 is important for *A. fumigatus*–associated phagocytosis and cytokine production (8, 9), and a premature stop codon polymorphism in Dectin-1 doubles the risk of invasive fungal disease in immunocompromised patients (11). Another CLR, Dectin-2, recognizes mannose-like structures in fungal cell walls, including that of *A. fumigatus*, and mediates important inflammatory responses upon fungal challenge (12–15). Mincle also recognizes mannans in fungal cell walls, and whereas Mincle expression is up-regulated in response to *A. fumigatus*, *A. fumigatus* did not activate Mincle-expressing cells (16, 17). SNPs in Dectin-1 and an additional CLR, dendritic cell-specific intercellular adhesion molecule-3-grabbing non-integrin (DC-SIGN), are associated with increased susceptibility to invasive aspergillosis. In addition, SNP–SNP interaction analysis demonstrated increased susceptibility in patients with SNPs in the genes that encode C-C motif chemokine ligand 2 (CCL2), Dectin-1, C-C motif chemokine receptor 2 (CCR2), and Dectin-2 (18).

Whereas defects in these receptors markedly increase susceptibility to fungal disease, little is known of their functional status in patients with underlying airway disease. Given the important complicating influence of fungal pathogens in CF, understanding the behavior of these receptors in the CF airway is of critical importance. Declining lung function in patients with CF is associated with a profound neutrophilia and the presence of high levels of free neutrophil-derived serine protease activity (19). Such proteases as neutrophil elastase (NE), cathepsin-G, and proteinase 3 have important antimicrobial functions, but are also capable of damaging host tissues and molecules of the immune system if not tightly regulated by the serpin family of antiproteases. A protease:antiprotease imbalance is widely recognized as an important pathologic mechanism in the CF airway (20). We and others have previously demonstrated that several arms of the immune system, including C5aR, IL-6, soluble IL-6R, surfactant protein-D, and TLRs (21–24), are subject to profound functional inactivation in the CF airway. Of note, Dectin-1 recognition of fungal ligands is trypsin sensitive (25), which raises the possibility that this critical fungal CLR may also be susceptible to inactivation by neutrophil-derived proteases in diseases such as CF. Here, we address this possibility and demonstrate that NE cleaves Dectin-1 and other CLRs, Dectin-2, and Mincle, which results in reduced antifungal responses.

## MATERIALS AND METHODS

### Mice

C57BL6/J, Dectin-1 knockout (KO) (26), Dectin-2 KO (27), Mincle KO (28), and BALB/c mice were maintained and handled according to institutional and UK Home Office guidelines. This study was performed in accordance with a project license approved by the Cardiff University Animal Welfare and Ethical Review Body and the UK Home Office. The animal care and use protocol adhered to the Animals (Scientific Procedures) Act 1986.

### Ethics and sample collection

Bronchoalveolar lavage (BAL) was collected from patients with CF who attended the Children’s Hospital for Wales. BAL samples from 35 patients are included in this study, with a median age at the time of sampling of 10.5 yr (interquartile range, 7–13.5 yr) and a gender distribution of 20:15 (female:male). Ethical approval for the collection and use of BAL samples in this study was obtained from the Wales Research Ethics Committee as part of the CF-SpIT study (11/WA/0334). BAL samples were obtained by the instillation and retrieval of normal saline at the right middle lobe by flexible bronchoscopy under general anesthetic as part of routine clinical care. BAL sampling was performed in accordance with the European Respiratory Society Task Force on BAL in children (29). In brief, a flexible bronchoscope was used to instill 0.9% sterile saline at of volume of 1 ml/kg to a maximum volume of 20 ml and immediately suctioned. Samples were stored at 4°C after collection and were processed in the laboratory within 1 h of collection. Samples were centrifuged at 500 *g* for 5 min at 4°C, and cell-free BAL fluid (BALF) was portioned into aliquots and stored at −80°C. For cytospins, cell pellets were resuspended in 10 mM EDTA/PBS and counted by using a hemocytometer. After cellular resuspension, 5 × 10^4^ cells were fixed to each polysine slide by using a cytofuge at 500 *g* for 5 min. For differential cell counts, cytospins were stained with hemacolor (EMD Millipore, Billerica, MA, USA) according to manufacturer’s instructions and fixed in DPX mounting medium. Cell differentiation was performed on at least 4 separate fields per cytospin at ×20 magnification, counting at least 300 events. Differential cell counts in recovered BAL from the 35 patients were as follows [median (interquartile range)]: total cell counts: 1.1 × 10^6^ cells/ml (0.76–2.56); polymorphonuclear cells: 0.83 × 10^6^ cells/ml (0.31–1.8); and mononuclear cells: 0.21 × 10^6^ cells/ml (0.11–0.31).

### Reagents

Elastase that was purified from primary human neutrophils was purchased from Athens Research and Technology (Athens, GA, USA). α-1 Antitrypsin (AAT) and PMSF were purchased from Sigma-Aldrich (St. Louis, MO, USA). Anti-hemagglutinin (HA) was purchased from Miltenyi Biotec (Bergisch Gladbach, Germany). Soluble recombinant human Dectin-1 was purchased from R&D Systems (Minneapolis, MN, USA). Anti–Dectin-1 (human and mouse) was purchased from Bio-Rad (Hercules, CA, USA). Anti-Ly6G, anti-CD11b, anti-F4/80, anti-CD19, and anti-TNF were purchased from BioLegend (San Diego, CA, USA). Anti-Mincle was purchased from Caltag MedBiosystems (Buckingham, United Kingdom). Anti–Dectin-2 mAb D2.11E4 has been previously described (30). Geneticin was purchased from Thermo Fisher Scientific (Waltham, MA, USA).

### Cell isolation and culture

NIH3T3 cell lines that expressed human or mouse Dectin-1 HA were a gift from Prof. Gordon Brown (University of Aberdeen, Aberdeen, United Kingdom). Cells were maintained in DMEM that was supplemented with 10% fetal bovine serum, 2 mM l-glutamine, and penicillin/streptomycin. Geneticin (G418) was added at a concentration of 0.4–0.6 mg/ml for selection. For passaging, cells were incubated with trypsin for 4–5 min at 37°C and detached by tapping the flask; however, for experiments, cells were removed from flasks by incubating with PBS that was supplemented with 10 mM EDTA and 4 mg/ml lidocaine (Sigma-Aldrich) to avoid receptor cleavage by trypsin. Medium was added to cells in trypsin or lidocaine and cells were centrifuged. Cells were washed twice in PBS before use.

To culture bone marrow–derived macrophages (BMDMs) (31), bone marrow cells were flushed from femurs and tibiae of mice. Bone marrow cells were resuspended at 2 × 10^5^ cells/ml in DMEM that was supplemented with 10% fetal bovine serum, 5% horse serum, 2 mM l-glutamine, penicillin/streptomycin, HEPES, and 10 ng/ml M-CSF. Cells were plated at a density of 7 × 10^6^ cells per 145-mm^2^ plate. Cells were cultured for 6–7 d, and 15 ml of fresh medium and M-CSF was added on d 3. Medium was removed from BMDM on d 6–7 and PBS that was supplemented with 8 mg/ml lidocaine was incubated with BMDMs for 4–5 min at 37°C. Cells were detached upon tapping and medium was added to cells. BMDMs were centrifuged and washed twice before use.

To recruit inflammatory cells, mice were injected intraperitoneally with 0.5 ml 2% (w/v) Biogel P-100 polyacrylamide beads (Bio-Rad) (32). Mice were sacrificed 16–18 h later, and inflammatory cells were collected by peritoneal lavage with 5 ml ice-cold RPMI 1640 that was supplemented with 10% fetal bovine serum and penicillin/streptomycin. Cells were passed through 40-µm cell strainers (BD Biosciences, San Jose, CA, USA) to remove Biogel beads. Cells were washed twice with assay buffer, PBS that was supplemented with 0.1% bovine serum albumin (BSA) and 5 mM EDTA, before use. Using Biogel injection, 9.37 × 10^6^ ± 0.80 × 10^6^ cells/mouse were elicited, and the cell population consisted of 71.68 ± 2.59% Ly6G^+^CD11b^+^ neutrophils and 15.48 ± 3.57% Ly6G^−^CD11b^+^ inflammatory monocytes.

### *Aspergillus* culture

*A. fumigatus* (CEA10 and Af293) strains were cultured on Sabouraud dextrose agar and incubated at 37°C for 48–72 h. Conidia were harvested with a PBS–Tween (0.05% Tween 20) wash. A flask that contained 500 ml Vogel’s minimal medium was inoculated with 1 × 10^6^ conidia/ml and cultured for 48 h at 37°C and 320 rpm. *A. fumigatus* cultures were then prefiltered through J cloth and sterile filtered through a 0.2-µm filter. Resultant culture filtrate was dialyzed overnight against repeated changes of distilled water and freeze dried before being stored at −80°C. Freeze-dried aliquots were reconstituted with sterile PBS, and total protein content was determined by using a BCA protein assay (Thermo Fisher Scientific). Total protease activity of culture filtrate was determined by using Universal Protease Substrate (Casein, resorufin labeled; Roche, Basel, Switzerland) according to manufacturer’s instructions. Filtrates were resuspended at 2 mg/ml in PBS. For CEA10, this was equivalent to 19 U/ml protease activity as measured by using the casein protease assay.

### Cleavage assays

For receptor cleavage experiments, cells were resuspended at 1–2 × 10^6^ cells/ml in assay buffer (PBS, 5 mM EDTA, 0.1% BSA, 10 mM NaN_3_). Cells (50 μl; 5–10 × 10^4^) were added to a 96-well plate and incubated with 50 µl of purified proteases in assay buffer or *A. fumigatus* filtrates at indicated concentrations for the indicated times at 37°C. For experiments that used CF BALF, cells were exposed to 50 µl of cell-free BALF for 30 min at 37°C. NE activity was neutralized either by using the indicated concentration of AAT or by heat inactivation (HI NE) at 95°C for 10 min. Cells were centrifuged, washed, and stained with anti-HA, anti–Dectin-1, anti–Dectin-2, or anti-Mincle. Biogel elicited cells were also stained with anti-Ly6G and anti-CD11b. Cells were then analyzed by flow cytometry on a Beckman Coulter CyAn, BD Canto, or FACScalibur flow cytometer (Beckman Coulter, Brea, CA, USA). Data were analyzed by using Flowing or FlowJo software (FlowJo, Ashland, OR, USA).

For experiments that examined the cleavage of soluble recombinant Dectin-1, 1 µg/ml of the recombinant protein in Tris-buffered saline (pH 7.4) was treated with the indicated concentration of NE or *A. fumigatus* filtrate for 60 min at 37°C. Reactions were stopped with the addition of 10 mM PMSF. Samples were subsequently prepared for SDS-PAGE under nonreducing conditions on a 15% Tris-glycine gel. Proteins were transferred to a nitrocellulose membrane, blocked with PBS-5% (w/v) milk, and probed with goat anti-human Dectin-1 (R&D Systems), followed by horseradish peroxidase conjugated donkey anti-goat IgG. Blots were visualized by using ECL radiography.

### NE activity assay

BALF was removed from −80°C and thawed on ice. BALF was diluted to <1:2 using activity buffer (0.1 M Tris, 0.5 M NaCl, 0.05% v/v Triton X-100, pH 7.5), and additional dilutions were made for samples with high activity. For standards, purified human NE was diluted 2-fold serially from 80 to 1.25 nM. Activity buffer was used as a negative control. BALF or standard (50 μl) was added to a flat-bottomed 96-well plate in duplicate. NE-specific chromogenic substrate (50 μl/well, 2mM), Suc-Ala-Ala-Pro-Val-pNA in activity buffer, was added. Optical density at 405 nm was read every minute for 20 min on a Dynex Revelation MRX TC spectrophotometer (Dynex Technologies, Chantilly, VA, USA). For each sample, the average change in optical density over time was calculated. NE activity in BALF was interpolated from a 7-point standard curve.

### Zymosan recognition assay

Zymosan (Thermo Fisher Scientific) was labeled with FITC (Sigma-Aldrich) as previously described (32). Labeled zymosan was washed 4 times with PBS and resuspended at 50 µg/ml in assay buffer ready for use. BMDMs or NIH3T3 cells that expressed human Dectin-1 isoform A or B were resuspended at 1 × 10^6^ cells/ml in assay buffer. Cells (50 μl; 5 × 10^4^) were added to a 96-well plate. Cells were centrifuged and supernatants were removed. Assay buffer, NE, or HI NE (50 μl) at the indicated concentrations were added for 1 h before the addition of 50 µl zymosan at 50 µg/ml for 15 min. Cells were centrifuged and washed. BMDMs were stained with anti-F4/80, anti-CD11b, and anti–Dectin-1. NIH3T3 cells that expressed human Dectin-1 isoform A or B were stained with anti–Dectin-1. Cells were analyzed by flow cytometry.

### Intracellular cytokine flow cytometry assay

*A. fumigatus* isolate 13073 (American Type Culture Collection, Manassas, VA, USA) swollen conidia (SC) were obtained by culturing 1 × 10^8^ *A. fumigatus* resting conidia in 1 ml RPMI 1640 and polymixin B (2 mg/ml) for 6 h at 37°C. SC were washed 3 times with PBS and labeled with 4.5 µM cell trace far red (Thermo Fisher Scientific) for 30 min at room temperature. SC were washed 3 times with PBS. Biogel elicited cells (100 μl; 4 × 10^5^) in RPMI 1640 that was supplemented with 10% fetal bovine serum and penicillin/streptomycin were added to a 96-well ultra-low adherence plate and left to rest for 1 h at 37°C. Medium or *A. fumigatus* SC (100 μl; 4 × 10^5^) was added for 3 h at 37°C in the presence of Brefeldin (BioLegend). Cells were stained with anti-Ly6G, anti-CD11b, anti-CD19, anti-TNF, and a live-dead stain (Thermo Fisher Scientific) and analyzed on a BD Canto flow cytometer. The following gating strategy was used to determine percent *A. fumigatus^+^* inflammatory monocytes producing TNF: live cells, CD19^−^, Ly6G^−^CD11b^+^, *A. fumigatus*^+^.

### Zymosan-induced ELISA cytokine assay

Biogel-elicited cells were resuspended at 8 × 10^6^ cells/ml in assay buffer. Cells (25 μl; 2 × 10^5^) were plated in a 96-well ultra-low adherence plate. Assay buffer, 4 µM NE, or HI NE (25 μl) was added for 1 h. Zymosan (50 μl; 50 µg/ml) was added for 4 h. NE and HI NE remained in the cultures during stimulation with zymosan. Cell culture supernatants were recovered and assayed for cytokine by ELISA (Thermo Fisher Scientific) according to the manufacturer’s protocol.

### *A. fumigatus*–induced ELISA cytokine assay

Biogel-elicited cells were washed with MACS buffer, PBS that was supplemented with 0.5% BSA and 2 mM EDTA, and stained with anti-Ly6G biotin (Miltenyi Biotec). Cells were incubated with anti-biotin microbeads (Miltenyi Biotec), and inflammatory monocytes (Ly6G^−^ cells) were enriched by MACS separation according to manufacturer’s instructions. Cells were resuspended in assay buffer, and 2–4 × 10^5^ cells were plated in a 96-well ultra-low adherence plate in 25 µl assay buffer. Assay buffer, 4 µM NE, or HI NE (25 μl) was added for 1 h. *A. fumigatus* SC (0.6–1.2 × 10^6^) in 50 µl Aim V medium was added for 4 h to stimulate cells at a 3:1 *A. fumigatus:*cell ratio. NE and HI NE remained in the cultures during stimulation with *A. fumigatus*. Cell culture supernatants were recovered and assayed for cytokine by ELISA according to manufacturer’s protocol.

### Statistical methods

Data were analyzed on GraphPad Prism (GraphPad Software, La Jolla, CA, USA). Data are presented as means ± sd or sem and are representative of 3–4 independent experiments. One-way ANOVA followed by Bonferroni’s posttest was used for statistical analysis when multiple groups were analyzed. A Student’s *t* test was used for statistical analysis when 2 groups were analyzed. A Spearman’s rank test was used to test correlations. Statistical significance was set at *P* < 0.05, *P* < 0.01, or *P* < 0.001.

## RESULTS

### Dectin-1A is cleaved by NE

**Figure 1.**
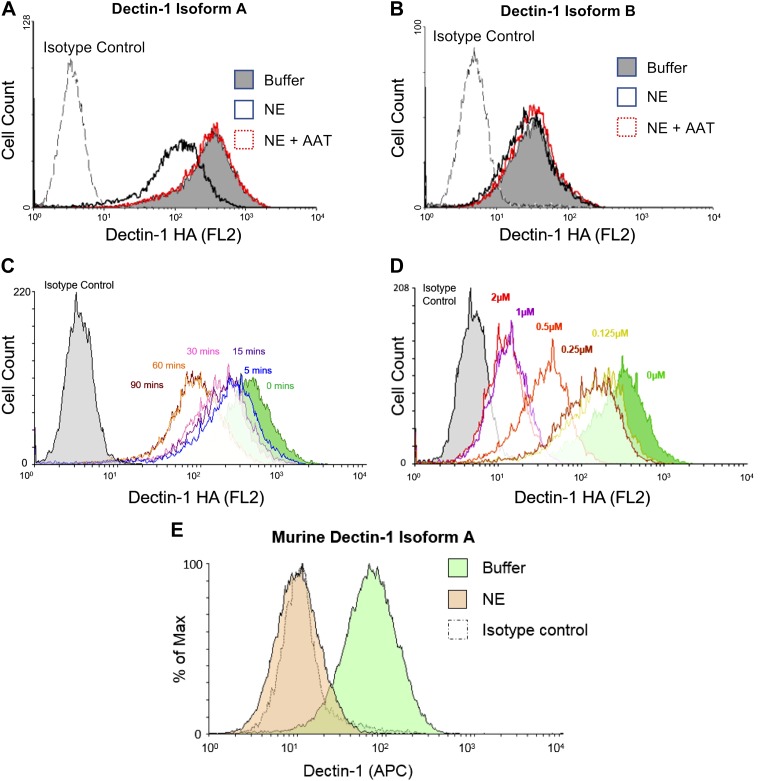
Dectin-1A is cleaved by NE. *A*–*C*) NIH-3T3 cells that express human HA-tagged Dectin-1 isoform A (*A*, *C*) or Dectin-1 isoform B (*B*) were exposed to 0.5 µM NE, in the presence or absence of AAT, for 30 min (*A*, *B*) or for the indicated times (*C*) at 37°C. Cells were stained with an anti-HA Ab and analyzed by flow cytometry. *D*) NIH-3T3 cells that express human HA-tagged Dectin-1 isoform A were exposed to the indicated concentrations of NE for 30 min at 37°C. Cells were stained with an anti-HA Ab and analyzed by flow cytometry. *E*) NIH-3T3 cells that express murine Dectin-1 isoform A were exposed to NE for 1 h at 37°C. Cells were stained with an anti–Dectin-1 Ab and analyzed by flow cytometry. Data are representative of 3–4 independent experiments. APC, allophycocyanin.

As NE cleaves various cell surface receptors, such as TLR4 and C5aR (23, 24), and Dectin-1 activity was trypsin sensitive (25), we postulated that NE may also cleave Dectin-1. To investigate this possibility, we incubated NIH3T3 cell lines that expressed 2 different isoforms of human Dectin-1 (hDectin-1) with NE. Dectin-1 isoform A is the full-length isoform, whereas isoform B lacks the stalk region (33). Of interest, NE only cleaved full-length Dectin-1 (isoform A; **Fig. 1*A***), but not the short isoform (isoform B; Fig. 1*B*), which suggests that the NE cleavage site is within the stalk region. NE-mediated cleavage of Dectin-1 was inhibited by the serine protease inhibitor, AAT (Fig. 1*A*). Similar to other receptors, NE-mediated cleavage of hDectin-1 isoform A occurred in a time- and dose-dependent manner (Fig. 1*C*, *D*). In addition to hDectin-1, NE also cleaved murine Dectin-1 isoform A (Fig. 1*E*). Thus, NE cleaves Dectin-1 isoform A, but not isoform B, which will likely have functional consequences in the presence of fungal pathogens.

### CF BALF induces cleavage of Dectin-1

**Figure 2.**
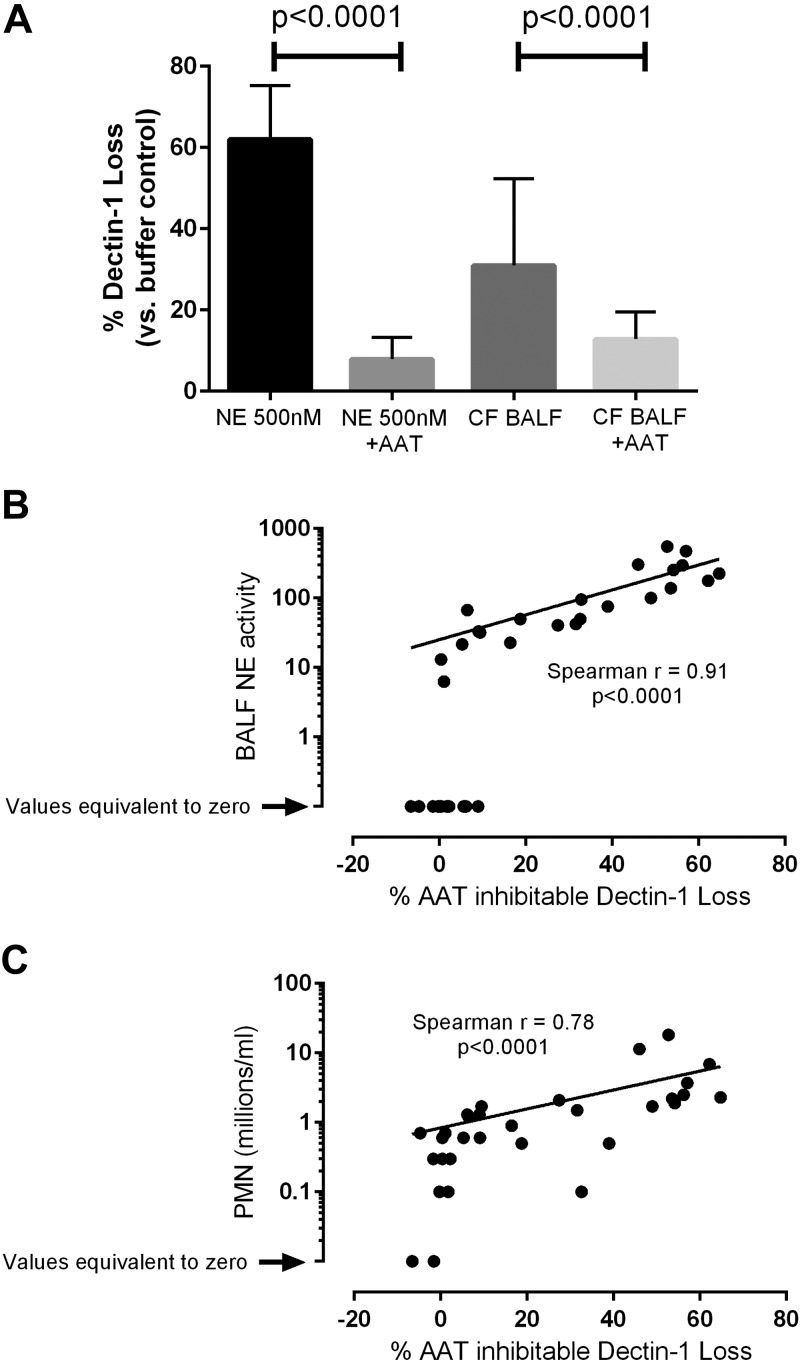
CF BALF induces cleavage of Dectin-1. *A*) NIH-3T3 cells that express human HA-tagged Dectin-1 isoform A were exposed to 0.5 µM NE or 50 µl CF BALF, in the presence or absence of AAT, for 30 min at 37°C. Cells were stained with anti-HA Ab and analyzed by flow cytometry. Graph displays means ± sd. Data are the cumulative result of 7 independent experiments. *B*, *C*) NIH3T3 cells that express human HA-tagged Dectin-1 isoform A were exposed to CF BALF for 30 min at 37°C. Cells were stained with an anti-HA Ab and analyzed by flow cytometry. NE activity in CF BALF samples was measured (*B*). Correlation analysis between NE activity and percentage loss of Dectin-1 was performed. Neutrophil numbers in the CF BALF were counted and correlation analysis between neutrophil numbers in CF BALF samples and percentage loss of Dectin-1 was performed (*C*). Graphs are the cumulative result of at least 7 independent experiments (*B*, *C*). Each symbol represents data for CF BALF from a single patient.

Increased neutrophil influx occurs in the lungs of patients with CF. This results in cellular activation, apoptosis, and necrosis, with a subsequent release of excess levels of NE (23). To determine whether BALF from the lungs of patients with CF has the ability to cleave Dectin-1, we incubated NIH3T3 cells that expressed hDectin-1 isoform A with either NE or BALF from patients with CF. Both NE and CF BALF substantially reduced cell surface Dectin-1 levels, an effect that was inhibited by the serine protease inhibitor, AAT (**Fig. 2*A***), thus demonstrating that CF BALF-induced cleavage of Dectin-1 was dependent on a serine protease. In addition to measuring the BALF-induced loss of Dectin-1, NE activity and neutrophil numbers in CF BALF samples were determined and examined for any relationship with the BALF-induced loss of Dectin-1 in the *in vitro* NIH-3T3 assay. A strong correlation was observed between NE activity and the loss of Dectin-1 (Fig. 2*B*), and between the number of neutrophils that are present in CF BALF and the loss of Dectin-1 (Fig. 2*C*). Although this does not rule out the possibility that other neutrophil serine proteases are also involved, these data indicate that Dectin-1 is cleaved by NE, and there is a strong association between Dectin-1 cleavage and neutrophil-mediated secretion of NE in the lungs of patients with CF.

### *A. fumigatus*–derived proteases cleave Dectin-1

**Figure 3.**
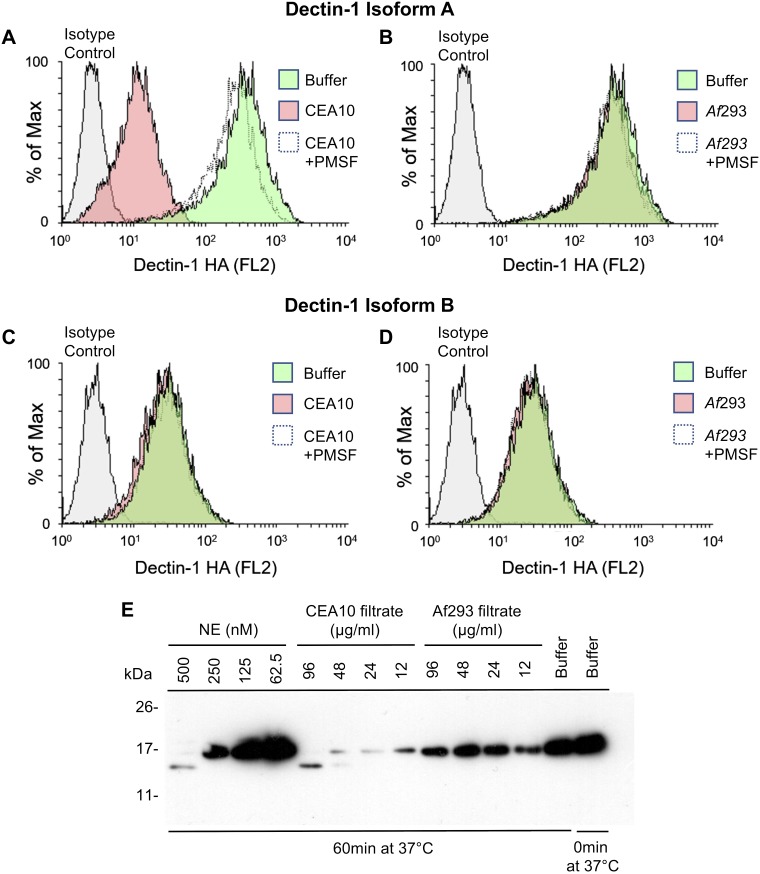
*A. fumigatus*–derived proteases cleave Dectin-1. *A*–*D*) NIH3T3 cells that express human HA-tagged Dectin-1 isoform A (*A*, *B*) or isoform B (*C*, *D*) were exposed to *A. fumigatus* CEA10 supernatant (*A*, *C*) or *Af293* supernatant (*B*, *D*)—in the presence or absence of PMSF—for 30 min at 37°C. Cells were stained with anti-HA Ab and analyzed by flow cytometry. Plots are representative of 3 independent experiments. *E*) Soluble Dectin-1 was exposed to NE or *A. fumigatus* filtrates from protease-producing (CEA10) or non–protease-producing (*Af293*) fungal strains at the indicated concentrations for 60 min at 37°C. Proteases were inhibited with PMSF, and Dectin-1 was separated by SDS-PAGE, blotted to nitrocellulose membrane, and probed with anti–Dectin-1. Data are representative of 3 independent experiments.

*A. fumigatus* has been isolated from the lungs of 10–57% of patients with CF, with increasing isolation from older patients and patients with worsening respiratory function (2). *A. fumigatus* secretes proteases, some of which display serine protease activity that is similar to NE (34). To investigate whether *A. fumigatus*–secreted proteases have the ability to cleave Dectin-1 in a manner similar to NE, we investigated 2 strains of *A. fumigatus*: CEA10, a high protease producer, and Af293, a low protease producer. Fungi were cultured in Vogel’s medium and the resulting culture filtrates were incubated with NIH3T3 cells that expressed hDectin-1. Culture filtrate (100 µg/ml total protein) from the high protease producer (*A. fumigatus* CEA10) induced the cleavage of hDectin-1 isoform A (**Fig. 3*A***), and this was inhibited by the serine protease inhibitor, PMSF; however, as expected, an equivalent quantity of culture filtrate from the low protease producer, *A. fumigatus* 293, did not induce the cleavage of hDectin-1 isoform A (Fig. 3*B*). Neither filtrate induced the cleavage of hDectin-1 isoform B (Fig. 3*C, D*). In addition to cleaving surface-bound Dectin-1, both NE and *A. fumigatus* CEA10 filtrate cleaved soluble Dectin-1, which resulted in a similar-sized fragment (Fig. 3*E*), whereas *A. fumigatus* 293 filtrate did not cleave soluble Dectin-1. These data indicate that, in addition to the NE-mediated cleavage of Dectin-1 in the lungs of patients with CF, *A. fumigatus*–secreted protease(s) may also cleave Dectin-1 in the lungs of patients with CF who are colonized with *A. fumigatus.*

### NE cleaves Dectin-1 on myeloid cells

**Figure 4.**
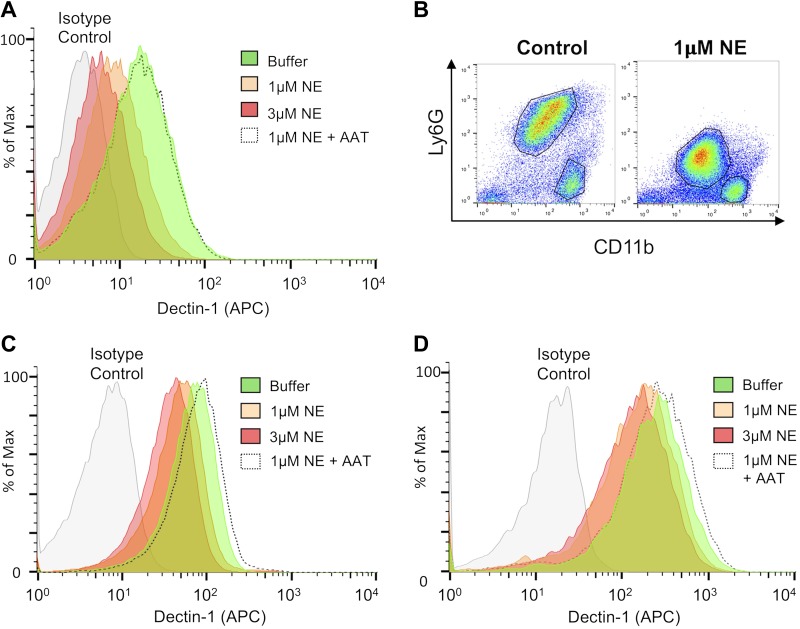
NE cleaves Dectin-1 on myeloid cells. *A*) BMDMs from BALB/c mice were exposed to NE, in the presence or absence of AAT, for 1 h at 37°C. Cells were stained with anti–Dectin-1 and analyzed by flow cytometry. *B*–*D*) BALB/c mice were injected i.p. with 0.5 ml Biogel, and inflammatory infiltrates were recovered 16 h later by peritoneal lavage. Cells were left unexposed or exposed to NE in the presence or absence of AAT for 1 h at 37°C. Cells were stained with anti-Ly6G, anti-CD11b, and anti–Dectin-1, and analyzed by flow cytometry. Dectin-1 levels were measured in the LyG^+^CD11b^+^ (neutrophil) population (*C*) and LyG^−^CD11b^+^ (inflammatory monocyte/macrophage) population (*D*). Data are representative of 3 independent experiments.

As we have shown that NE cleaves Dectin-1 on NIH3T3 cells that overexpress Dectin-1, we undertook experiments to determine whether this occurs on myeloid cells that naturally express Dectin-1. Heinsbroek *et al.* (33) demonstrated that macrophages from BALB/c mice expressed similar levels of Dectin-1 isoform A and isoform B, whereas macrophages from C57BL/6 mice predominantly expressed isoform B; therefore, as only the Dectin-1A isoform is cleaved by NE, we examined NE-mediated Dectin-1 cleavage on myeloid cells from BALB/c mice. Consistent with our findings with NIH3T3 cells that overexpressed Dectin-1, NE cleaved Dectin-1 on BMDMs from BALB/c mice in a dose-dependent manner, and this was inhibited by the serine protease inhibitor, AAT (**Fig. 4*A***). As neutrophils and inflammatory cells are recruited to the lungs of patients with CF, we elicited neutrophils and inflammatory monocytes/macrophages by injecting Biogel into the peritoneal cavity. These 2 populations were clearly identifiable as Ly6G^+^CD11b^+^ neutrophils and Ly6G^−^CD11b^+^ inflammatory monocytes/macrophages in the absence of NE treatment (Fig. 4*B*). After treatment with NE for 1 h, the 2 populations could still be identified by using these markers; however, Ly6G expression was substantially reduced (Fig. 4*B*), which indicated NE-mediated cleavage of Ly6G. NE also induced Dectin-1 cleavage in Biogel-elicited neutrophils (Fig. 4*C*) and inflammatory monocytes/macrophages (Fig. 4*D*) in a dose-dependent manner, and this was inhibited by AAT; therefore, NE cleaves Dectin-1 from the surface of various myeloid cell populations.

### NE cleavage of Dectin-1 impairs zymosan recognition

**Figure 5.**
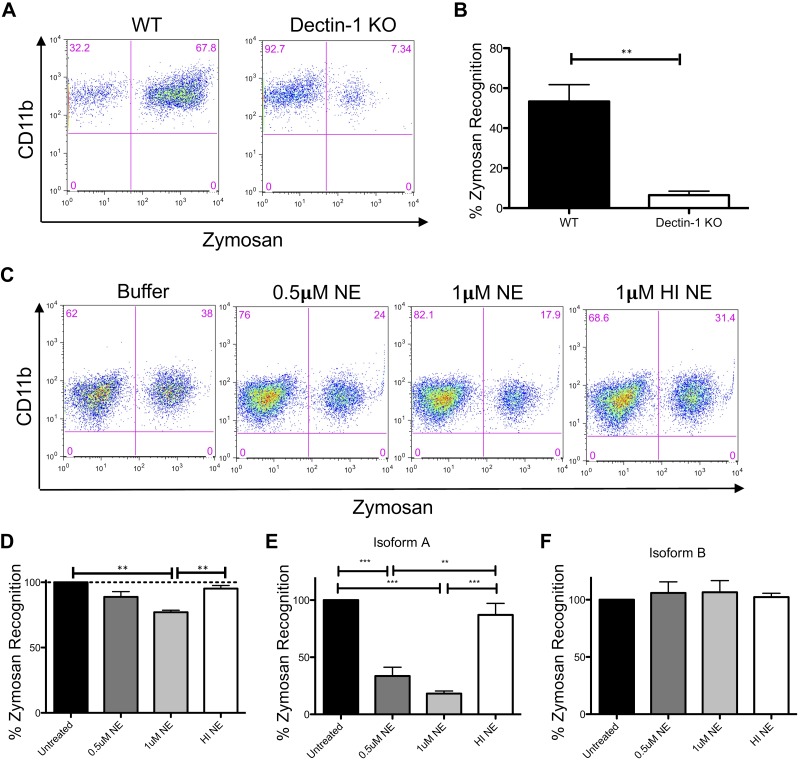
NE cleavage of Dectin-1 impairs zymosan recognition. *A*–*D*) Wild-type (WT) and Dectin-1 KO BMDMs (*A*, *B*) and NE-treated BMDMs from BALB/c mice (*C*, *D*) were incubated with 2.5 µg FITC-labeled zymosan for 15 min. Cells were stained with anti-F4/80, anti-CD11b, and anti–Dectin-1, and analyzed by flow cytometry. Data are representative of 3 independent experiments. Zymosan recognition in Dectin-1 KO cells (*B*) and NE/HI NE-treated cells (*D*) is relative to WT cells (set at 100%; *B*) or buffer control cells (set at 100%; *D*). *E*, *F*) NE-treated NIH3T3 cells that express hDectin-1 isoform A (*E*) or isoform B (*F*) were incubated with 2.5 µg FITC-labeled zymosan for 15 min. Cells were stained with anti–Dectin-1 and analyzed by flow cytometry. Data are representative of 3 independent experiments. Zymosan recognition in NE/HI NE-treated cells is relative to buffer control cells (set at 100%). Graphs display means ± sem. Representative data from 1 of the 3 independent experiments (*A*, *C*). **P < 0.01, Student’s *t* test (*B*); **P < 0.01, ***P < 0.001, 1-way ANOVA with Bonferroni’s posttest (*D*, *E*).

Dectin-1 is a phagocytic receptor for various fungal pathogens (9). To determine whether NE-induced cleavage of Dectin-1 resulted in the reduced recognition of β-glucan–containing substrates, we used the fungal cell wall preparation, zymosan, which is rich in β-glucans. In agreement with previous findings, we showed that Dectin-1 KO BMDMs displayed significantly reduced zymosan recognition (**Fig. 5*A, B***). In addition, BMDMs from BALB/c mice that were treated with NE demonstrated reduced zymosan recognition in a dose-dependent manner (Fig. 5*C, D*). HI NE did not substantially reduce zymosan recognition (Fig. 5*C, D*). Similarly, NE treatment of NIH3T3 cells that expressed hDectin-1 isoform A resulted in reduced zymosan recognition in a dose-dependent manner (Fig. 5*E*); however, NE treatment of NIH3T3 cells that expressed hDectin-1 isoform B did not affect zymosan recognition (Fig. 5*F*). This is consistent with the inability of NE to cleave hDectin-1 isoform B (Fig. 1*B*). Therefore, reduced Dectin-1 expression as a result of NE-induced cleavage in the lungs of patients with CF would likely result in the reduced recognition of fungal pathogens that are rich in β-glucans.

### NE cleaves CLRs and reduces TNF production

**Figure 6.**
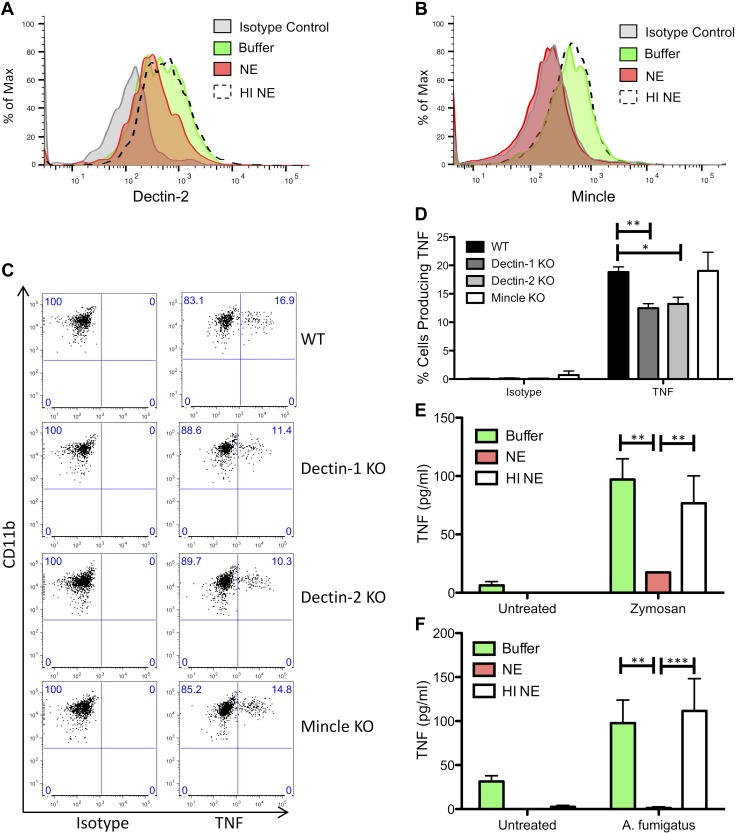
NE cleaves CLRs and reduces TNF production. *A*, *B*) BALB/c mice were injected with Biogel (0.5 ml, i.p.), and inflammatory infiltrates were recovered 16 h later by peritoneal lavage. Cells were left unexposed or exposed to 1 µM NE or HI NE for 1 h at 37°C. Cells were stained with anti-Ly6G, anti-CD11b, anti–Dectin-2, and anti-Mincle, and analyzed by flow cytometry. Dectin-2 (*A*) and Mincle (*B*) levels were measured in the LyG^−^CD11b^+^ inflammatory monocyte/macrophage population. *C*, *D*) Wild-type (WT), Dectin-1 KO, Dectin-2 KO, and Mincle KO mice were injected intraperitoneally with 0.5 ml Biogel, and inflammatory infiltrates were recovered 16 h later by peritoneal lavage. Cells were stimulated with cell trace far red-labeled *A. fumigatus* for 3 h in the presence of Brefeldin, and cells were stained with anti-Ly6G, anti-CD11b, anti-CD19, and anti-TNF, and analyzed by flow cytometry. Percentages of TNF-producing inflammatory monocytes with bound/phagocytosed *A. fumigatus* were measured. Representative data from 1 of 3 independent experiments is shown in panel *C*. Graph displays means ± sem (*D*). Graph displays cumulative data from 3 independent experiments. *P < 0.05, **P < 0.01, 2-way ANOVA with Bonferroni’s posttest. *E*) BALB/c mice were injected with Biogel (0.5 ml, i.p.), and inflammatory infiltrates were recovered 16 h later by peritoneal lavage. Cells were left unexposed or exposed to 2 µM NE or HI NE for 1 h at 37°C, followed by stimulation with 25 µg/ml zymosan for 4 h. TNF levels in the supernatants were measured by ELISA. Graph displays means ± sem. Graph displays cumulative data from 3 independent experiments. **P < 0.01, 2-way ANOVA with Bonferroni’s posttest. *F*) BALB/c mice were injected with Biogel (0.5 ml, i.p.), and inflammatory infiltrates were recovered 16 h later by peritoneal lavage. The inflammatory monocyte population was enriched by Ly6G^+^ cell depletion. Cells were left unexposed or exposed to 2 µM NE or HI NE for 1 h at 37°C, followed by stimulation with *A. fumigatus* for 4 h. TNF levels in the supernatants were measured by ELISA. Graph displays means ± sem. Graph displays cumulative data from 4 independent experiments. **P < 0.01, ***P < 0.001, paired 2-way ANOVA with Bonferroni’s posttest.

As we have shown that Dectin-1 is cleaved by NE, we conducted experiments to determine whether additional CLRs, such as Dectin-2 or Mincle, were also cleaved by NE. To this end, Biogel-elicited cells were treated with NE, and Dectin-2 and Mincle expression levels were determined by flow cytometry. Similar to our findings with Dectin-1, NE cleaved both Dectin-2 and Mincle on inflammatory monocytes/macrophages (**Fig. 6*A, B***) and neutrophils (data not shown). *A. fumigatus*–induced TNF production was reduced in inflammatory monocytes/macrophages from Dectin-1 KO and Dectin-2 KO mice, but not Mincle KO mice (Fig. 6*C, D*). In addition, zymosan-induced TNF production from Biogel-elicited cells was reduced after incubation with NE, whereas HI NE did not substantially reduce TNF production (Fig. 6*E*). Similarly, *A. fumigatus*–induced TNF production from an enriched population of Biogel-elicited inflammatory monocytes was reduced after treatment with NE, whereas HI NE did not reduce TNF production (Fig. 6*F*). As both Dectin-1 and Dectin-2 contribute to fungal-induced TNF production, and as both CLRs are cleaved by NE, NE-mediated blockade of TNF production is likely a result, in part, of the loss of Dectin-1 and Dectin-2, although contributing roles for other receptors, such as TLRs, cannot be ruled out.

## DISCUSSION

Here, for the first time, we have demonstrated that Dectin-1 is cleaved by NE in an isoform-specific manner, and we have shown that BALF from patients with CF also induces Dectin-1 cleavage. The percentage loss of Dectin-1 at the surface of cells *in vitro* correlates with NE activity and neutrophil numbers in BALF. We also determined that culture filtrate from a high protease-producing strain of *A. fumigatus* induces the cleavage of Dectin-1 in an isoform-specific manner, similar to that observed with NE treatment. In addition, Dectin-1 KO BMDMs and NE-treated BMDMs demonstrated reduced recognition/phagocytosis of zymosan, a fungal cell wall preparation. Finally, NE cleaves two other CLR family members, Dectin-2 and Mincle, and fungal-induced TNF production was reduced in Dectin-1 KO cells, Dectin-2 KO cells, and NE-treated cells; therefore, cleavage of Dectin-1 and Dectin-2 by NE and/or *A. fumigatus*–derived proteases results in an aberrant antifungal immune response. Taken together, these data indicate that both host-derived NE and *Aspergillus*-derived proteases have the capacity to cleave Dectin-1 and other CLRs in the lungs of patients with CF, potentially inhibiting immune responses to *Aspergillus*, thereby reducing the ability to clear *Aspergillus* from the airway.

CF affects ∼1 in 2500 live births in the United Kingdom. Although it is a multiorgan disease, the majority of morbidity and mortality is associated with progressive airway disease as a result of recurrent and chronic infection. The neutrophils that dominate the airway during disease exacerbation, and the proteases they secrete, are primary contributors to declining airway function in CF (20). In addition to their damaging effects on the mucocilliary system and other local tissue structures, neutrophil serine proteases (NSPs) cleave and inactivate several important components of the immune system, which compromises effective immunity. For example, we demonstrated that NE and other NSPs in BALF from patients with CF cleave and inactivate C5aR (23). Of interest, another study demonstrated that TLR4 is cleaved by NE; however, that study showed that NE promotes cytokine production mediated, in part, by TLR4 (24). This is in contrast to our results with Dectin-1; however, this may be a result of different experimental conditions. That previous study incubated cells with NE for 1 h, then removed NE before additional culture for 4 h (24). In contrast, we incubated cells with NE for 1 h and added zymosan for an additional 4 h in the presence of NE. This resulted in diminished cytokine production in response to zymosan in the presence of NE, which indicated an aberrant response to *A. fumigatus* in settings in which high levels of NE are present for a prolonged time. Whereas CF lungs are frequently exposed to high levels of NE and other NSPs, and they express high levels of proinflammatory cytokines, there are likely multiple factors that are involved in the induction of these proinflammatory cytokines. On the basis of our data, we hypothesize that exposure to *A. fumigatus* in patients with CF who undergo an airway exacerbation may lead to aberrant antifungal immune responses as a result of protease-mediated deficiency of Dectin-1 and other antifungal receptors. This may result in an inability to effectively clear *A. fumigatus* from the lungs, and may also lead to sensitization to *A. fumigatus* and the development of ABPA.

Whereas much of our data focus on the potential for neutrophil-derived proteases to functionally inactivate Dectin-1, we also present data that show that proteases expressed by *A. fumigatus* itself have the potential to inactivate this important fungal receptor. A wide range of fungal species are known to express proteases, and these are thought to play important roles in normal fungal growth and propagation as well as facilitating tissue invasion and catabolizing extracellular macromolecules as a food source (35). Proteases that are secreted by *A. fumigatus* are also recognized as important antigens that mediate allergic immune responses. Whereas there is little evidence to suggest that they act in a directly immune-evasive manner, a previous report has demonstrated their capacity to cleave the innate immune pattern recognition receptor, Pentraxin-3 (34). Data presented in this study indicate that these proteases may further benefit the pathogen *via* inactivation of fundamental antifungal innate immune receptors. As *A. fumigatus* secretion of proteases is strain dependent, *A. fumigatus* protease-dependent Dectin-1 cleavage may vary between patients, depending on which strain(s) of *A. fumigatus* are present. Additional studies that focus on *in vivo* models with protease-sufficient and -deficient fungal strains will be required to fully understand the importance of this observation to fungal pathogenesis.

CLRs, Dectin-1, Dectin-2, and Mincle are important for antifungal responses to *A. fumigatus* and/or other fungal spp. The immunologically inert surface RodA layer on resting *A. fumigatus* conidia masks other immunologically active cell wall components, such as β-glucans and mannans. Upon removal of this layer by swelling/germination, host immune pathogen recognition receptors recognize cell wall components and induce an immune response (36). In agreement with this, Dectin-1 does not bind *A. fumigatus* resting conidia, but binds to SC, early germlings, and hyphae (9, 10). Dectin-1 is important for phagocytosis of fungal pathogens, and we observed reduced zymosan recognition/binding by Dectin-1 KO BMDMs and NE-treated BMDMs, with reduced Dectin-1 expression. *A. fumigatus* SC and early germlings induce a robust cytokine response that is mediated, in part, by Dectin-1 and TLR2 (8–10). In addition, Dectin-2 is partially responsible for *A. fumigatus* SC and hyphae-induced reactive oxygen species and/or cytokine production (14, 37). Similar to Dectin-1–induced responses, Dectin-2–mediated responses are blocked by the presence of the RodA layer on resting conidia (12). Whereas Mincle expression is up-regulated in response to *A.* *fumigatus* stimulation (16), a role for Mincle during the response to *A. fumigatus* has not been identified to date (17). In agreement with these data, we observed that *A. fumigatus* SC-induced TNF from Biogel-elicited inflammatory monocytes/macrophages was partially dependent on Dectin-1 and Dectin-2, but independent of Mincle. This does not rule out the involvement of other receptors, such as TLR2, which has previously been shown to play a role in *A. fumigatus*–induced cytokine production from macrophages (9, 10). NE-treated cells, with reduced Dectin-1, Dectin-2, and Mincle expression, also displayed reduced fungal-induced cytokine responses, which is in agreement with the reduced cytokine responses that we observed in Dectin-1 and Dectin-2 KO cells. Therefore, CLRs, Dectin-1, and Dectin-2 are involved in the antifungal response to *A. fumigatus*, and loss of these receptors as a result of protease-induced cleavage results in an aberrant response.

In addition to pathogen recognition and cytokine production by innate immune cells, TLRs and/or CLRs are involved in inflammatory cell recruitment, fungal killing, and the induction of T-cell responses. Werner *et al.* (8) demonstrated that Dectin-1 KO mice displayed reduced neutrophil recruitment compared with wild-type mice in a pulmonary model of *Aspergillus* infection. Another study demonstrated reduced inflammatory cell recruitment in Dectin-1 KO mice during a corneal infection model with *Aspergillus*, whereas cell recruitment was unimpaired in TLR2 KO or TLR4 KO mice; however, fungal killing was impaired in TLR4 KO mice (38). *A. fumigatus* induces both T helper (T_h_)1 and T_h_17 responses, which depend on TLR/MyD88 and Dectin-1 signaling, respectively. Dectin-1 signals reduce IL-12 and IFN-γ production in innate cells, which results in decreased T-bet expression in *A. fumigatus*–specific CD4 T cells, thereby facilitating T_h_17 differentiation (39). Dectin-2 is important for the induction of T_h_17 responses to *Candida albicans* (27, 40), and *A. fumigatus*–induced Dectin-2 signaling promotes IL-1β and IL-23 production (37), which suggests that it will also be involved in the induction of *A. fumigatus*–associated T_h_17 responses. We could speculate that NE-induced cleavage of these CLRs, and potentially TLR4, could reduce T_h_17 and T_h_1 responses, leading to increased Th2 responses like that observed in ABPA; however, additional study is required to determine if this is the case. The isoform-specific susceptibility of Dectin-1 to such proteolysis is intriguing. The functional relevance of the 2 main human isoforms of Dectin-1 (A and B) remains unclear, although it is known that they are differentially expressed by myeloid subsets and at different sites, and recent evidence demonstrates distinct inflammatory responses to the ligation of either isoform (41). As isoform B seems to be resistant to proteolysis, we speculate that this isoform may play a particularly important role in antifungal immunity during periods of intense neutrophilic inflammation.

It may be difficult to establish whether the protease-mediated reduction of Dectin-1 expression on airway cells correlates with the incidence of APBA and/or *A. fumigatus* colonization in the human population. This is a result of the fact that patient BAL samples are collected intermittently and may not coincide with the original sensitization or colonization event. In addition, the patient cohort under investigation in this study is relatively small and is derived from a pediatric population in whom fungal pathology is less prevalent than in older patients with CF (3).

Overall, our data suggest a novel role for neutrophil and fungal-derived proteases in the modulation of host immune responses to fungal pathogens. We have demonstrated the functionally relevant and isoform-specific inactivation of Dectin-1 and other fungal receptors by these proteases in both man and mouse. Additional studies are required to shed light on the pathologic importance of these observations in various diseases in which excess protease activity is observed.

## Abbreviations

AAT: α-1 antitrypsin
ABPA: allergic bronchopulmonary aspergillosis
BAL: bronchoalveolar lavage
BALF: bronchoalveolar lavage fluid
BMDM: bone marrow–derived macrophages
BSA: bovine serum albumin
CF: cystic fibrosis
CLR: C-type lectin-like receptor
hDectin-1: human Dectin-1
HA: hemagglutinin
HI NE: heat-inactivated neutrophil elastase
KO: knockout
NE: neutrophil elastase
NSP: neutrophil serine protease
SC: swollen conidia
SNP: single nucleotide polymorphism
T_h_: T helper
